# Cannabidiol promotes intestinal cholesterol uptake mediated by Pregnane X receptor

**DOI:** 10.3389/fendo.2024.1398462

**Published:** 2024-06-18

**Authors:** Conner Brown, Wangeci Kariuki, Haizhen A. Zhong, Audra Kippes, Yipeng Sui

**Affiliations:** ^1^ Department of Biology, University of Nebraska at Kearney, Kearney, NE, United States; ^2^ Department of Chemistry, University of Nebraska at Omaha, Omaha, NE, United States

**Keywords:** cannabidiol, Pregnane X receptor, cholesterol uptake, dyslipidemia, cardiovascular disease

## Abstract

**Background:**

Cannabidiol (CBD), a non-psychoactive phytocannabinoid of cannabis, is therapeutically used as an analgesic, anti-convulsant, anti-inflammatory, and anti-psychotic drug. There is a growing concern about the adverse side effects posed by CBD usage. Pregnane X receptor (PXR) is a nuclear receptor activated by a variety of dietary steroids, pharmaceutical agents, and environmental chemicals. In addition to the role in xenobiotic metabolism, the atherogenic and dyslipidemic effects of PXR have been revealed in animal models. CBD has a low affinity for cannabinoid receptors, thus it is important to elucidate the molecular mechanisms by which CBD activates cellular signaling and to assess the possible adverse impacts of CBD on pro-atherosclerotic events in cardiovascular system, such as dyslipidemia.

**Objective:**

Our study aims to explore the cellular and molecular mechanisms by which exposure to CBD activates human PXR and increases the risk of dyslipidemia.

**Methods:**

Both human hepatic and intestinal cells were used to test if CBD was a PXR agonist via cell-based transfection assay. The key residues within PXR’s ligand-binding pocket that CBD interacted with were investigated using computational docking study together with site-directed mutagenesis assay. The C57BL/6 wildtype mice were orally fed CBD in the presence of PXR antagonist resveratrol (RES) to determine how CBD exposure could change the plasma lipid profiles in a PXR-dependent manner. Human intestinal cells were treated with CBD and/or RES to estimate the functions of CBD in cholesterol uptake.

**Results:**

CBD was a selective agonist of PXR with higher activities on human PXR than rodents PXRs and promoted the dissociation of human PXR from nuclear co-repressors. The key amino acid residues Met246, Ser247, Phe251, Phe288, Trp299, and Tyr306 within PXR’s ligand binding pocket were identified to be necessary for the agonistic effects of CBD. Exposure to CBD increased the circulating total cholesterol levels in mice which was partially caused by the induced expression levels of the key intestinal PXR-regulated lipogenic genes. Mechanistically, CBD induced the gene expression of key intestinal cholesterol transporters, which led to the increased cholesterol uptake by intestinal cells.

**Conclusion:**

CBD was identified as a selective PXR agonist. Exposure to CBD activated PXR signaling and increased the atherogenic cholesterol levels in plasma, which partially resulted from the ascended cholesterol uptake by intestinal cells. Our study provides potential evidence for the future risk assessment of CBD on cardiovascular disease, such as dyslipidemia.

## Introduction

Plants have been used in anti-asthmatic, anti-rheumatic, and analgesic treatment. Cannabis contains over 700 chemicals among which a group of Cannabinoids compounds, phytocannabinoids, attract more concerns (1, 2). Two well-known phytocannabinoids are tetrahydrocannabinol (THC) and cannabidiol (CBD). The adverse effects of THC have been established (3), therefore, medical cannabis patients frequently use CBD predominant products with the minimal amounts of THC to have the optimal improvement in symptoms and quality of life (4). CBD as a non-psychoactive phytocannabinoid of cannabis (1, 2) is therapeutically used as an analgesic, anti-convulsant, anti-inflammatory, and anti-psychotic drug (5, 6). Since 2014, CBD has been marketed to treat physical ailment (7). However, there is growing concern about the adverse side effects posed by CBD usage (8). For example, marijuana smoking has been associated with cardiovascular complications, such as tachycardia and acute coronary events (9).

CBD is transported through the bloodstream and distributed to the blood-abundant organs, e.g., heart, lung, and liver, chronically accumulating in adipose tissue due to the high lipophilicity (10). The research in both humans and animals has suggested that cannabinoids could affect cardiovascular system (11, 12). CBD treated microglial cells displayed the upregulated mRNA level of the cholesteryl esters synthesis enzymes and lipid droplet-associated protein, suggesting the possible regulation of CBD on cholesterol homeostasis in microglial cells (13). A recent study also suggested that CBD injection increased lipid peroxidation and free fatty acid levels in rats (14). Cannabinoid receptors and the corresponding metabolizing enzymes are identified in the cardiovascular system. Interestingly, the affinity of THC to cannabinoid receptors is high, whereas CBD has a low affinity for cannabinoid receptors (15). Thus, it is critically important to elucidate the molecular mechanisms by which CBD activates cellular signaling and to assess the possible adverse impacts of CBD on pro-atherosclerotic events in the cardiovascular system, such as dyslipidemia.

Previous studies have identified the dyslipidemic effects of several clinically used drugs mediated by pregnane X receptor (PXR; also known as steroid and xenobiotic receptor, or SXR) (16–20). Pregnane X receptor (PXR) is a nuclear receptor activated by a variety of dietary steroids, pharmaceutical agents, and environmental chemicals (16, 19, 21). In addition to the role in xenobiotic metabolism, the atherogenic effect of PXR has been revealed in animal models (21, 22). The chronic activation of PXR increases atherosclerosis in mice by increasing macrophage lipid accumulation and atherosclerotic foam cell formation. Many clinically relevant PXR ligands, including rifampicin, cyclosporine A, and carbamazepine, can elevate plasma lipid levels in patients and increase their cardiovascular disease (CVD) risk. For example, Quetiapine, one of the most prescribed second-generation (atypical) antipsychotics for the treatment of several psychiatric conditions, is established to stimulate hypercholesterolemia in mice through increased PXR-mediated intestinal lipid absorption (23). PXR signaling is involved in the regulation of lipid homeostasis, therefore, PXR is an important tool to study pharmaceutical agent influenced disease (18, 20, 24–28). However, it is still unknown whether CBD can activate PXR signaling.

We hypothesized that CBD could promote intestinal cholesterol uptake by activating PXR signaling leading to increased plasma cholesterol levels. We used both human hepatic and intestinal cells to test if CBD was a PXR agonist and investigated the key residues within PXR’s ligand binding pocket that CBD interacted with using computational docking study together with site-directed mutagenesis assay. The C57BL/6 wildtype mice were orally fed CBD in the presence of PXR antagonist resveratrol (RES) to determine how CBD exposure could change the plasma lipid profiles *in vivo* in a PXR-dependent way. Mechanistically, human intestinal cells were treated with CBD and/or RES to estimate the functions of CBD in cholesterol uptake. Our study aims to explore the cellular and molecular mechanisms by which exposure to CBD activates human PXR and increases the risk of dyslipidemia.

## Materials and methods

### Reagents and plasmids

CBD (Cayman Chemical, 90080) and RES (TCI Chemicals, R0071) were dissolved in dimethyl sulfoxide (DMSO). The plasmids used in this study were previously described, including human (h) and mouse (m) PXR expression vectors (16, 29), PXR-dependent CYP3A4 promoter reporter (CYP3A4XREM-Luciferase) (30, 31) and CYP3A2 promoter reporter ((CYP3A2)_3_-luciferase) (16, 29), β-galactosidase (β-gal) expression vector (16, 32); GAL4 DNA-binding domain (DBD)-linked nuclear receptor ligand binding domain (LBD) vectors (31, 33, 34), VP16-hPXR (31, 33), GAL4 DBD-linked nuclear receptor co-repressors (SMRT, Silencing mediator of retinoid and thyroid hormone receptors; NCoR, Nuclear receptor co-repressor) (31, 33, 35), and GAL4 reporter (MH100-Luciferase) (16, 31).

### Cell culture and transfection assay

The human hepatic cell line HepG2 (ATCC, HB-8065) and human intestine epithelial cell line LS180 (ATCC, CL-187) was obtained from American Type Culture Collection and cultured in DMEM containing 10% FBS at 37°C in 5% CO_2_. Transfection assays were performed as previously described (36). The cells with 80% confluency in 24-well plates were transiently transfected with various expression plasmids as well as the corresponding luciferase reporter plasmids, together with β-gal control plasmids using FuGENE 6 (Promega Corporation, E2691) in serum-free DMEM. For mammalian two-hybrid assays, HepG2 cells were transfected with MH100-Luciferase GAL4 reporter, VP16-hPXR, and co-repressor plasmids (33, 35) for 24 hours, and were treated with chemicals or DMSO vehicle for 24 hours in serum-free DMEM. The cell lysate was prepared for luciferase and β-galactosidase assays. Luciferase assays were performed as manufacturer’s manual (Promega Corporation, E1531) using a Synergy H1 Hybrid Reader (Agilent BioTek Instruments, 11120533). For the β-galactosidase assays the cell lysate was added with β-gal solution and incubated at 37 °C for 30 min, and then the reaction was stopped with 1M Na_2_CO_3_. The lysate was then read OD_420_ using a Synergy H1 Hybrid Reader. Reporter gene activity was normalized to the β-gal transfection controls and the results expressed as normalized Relative Light Unit (RLU) per OD_420_ β-gal per minute to facilitate comparisons between plates. Fold activation was calculated relative to solvent controls. EC_50_ values were calculated by curve fitting of data, using GraphPad Prism software.

### Computational docking studies

The structural coordinates of the human PXR with bound ligand (17-α-ethinylestradiol) were retrieved from the Research Collaboratory for Structural Bioinformatics (RCSB) Protein Data Bank (PDB id: 4X1F) (37). There were two gaps with residues missing coordinates on the crystal structure 4X1F and the gaps were fixed using the Loop Modeler module in the Molecular Operating Environment (MOE) program (Chemical Computing Group Inc., Montreal, QC, Canada), followed by energy minimization using the AMBER12:EHT force field, first on the side chain residues and then on the whole system (38). The resulting protein structural coordinates were saved in the pdb format and imported to Maestro program for the Glide Dock study using the Schrödinger software suite (Schrödinger LLC., New York, NY, USA). After importing to the Maestro software interface, the protein with the bound ligand was further prepared using the Protein Preparation Wizard in the Schrödinger software suite followed by energy minimization using the OPLS3 force field first by restraining the backbone then by fully optimizing the whole system using the MacroModel module as implemented in the Schrödinger software (39).

To investigate the mutational effect on binding residues, we also prepared mutation models based on the results of the above Glide Dock study. The mutant proteins were prepared using the same protocol as the wildtype PXR protein (4X1F wt). The ligand structure of CBD was constructed in the MOE program and was saved and imported to the Maestro for docking studies. The bound ligand in the model proteins 4X1F wt, Met243Ala, Met246Ala, Ser247Leu, Phe251Leu, Gln285Ala, Phe288Ala, Trp299Leu, and Tyr306Phe were used as the centroid to define the binding pocket using the Schrödinger Glide Grid Generation program, followed by docking the ligand CBD to the binding pocket as defined by the generated grid files. During the Glide Dock process, the scoring function of Extra-Precision was used and everything else used the default parameters. After docking, the binding affinity for the protein/docked ligand interaction was given in Glide Score in kcal/mol. The protein-ligand interaction figure was made by Pymol program (DeLano Scientific, San Carlos, CA, USA) (40).

### Site-directed mutagenesis

Human PXR full-length plasmid was used as a wildtype template to generate a series of mutant plasmids by utilizing QuikChange II Site-Directed Mutagenesis Kit according to the manufacturer-supplied protocol (Agilent, 200524). The primers used for mutant generation are listed in **Supplementary Table 1**.

### Animals

Wildtype C57BL/6 male mice were purchased from Taconic Biosciences Inc (Germantown, NY). In a recent rat study CBD injection at a dose of 10 mg/kg body weight (BW) daily for 14 days was identified to increase lipid peroxidation and free fatty acid levels (14). Previously we used three different doses (2.5, 5, or 10 mg/kg/day) of another PXR selective agonist tributyl citrate to treat mice for 7 days, and the results suggested that only the dose of 10 mg/kg/day could activate intestinal PXR (28). To determine the optimal CBD dose that could activate PXR signaling in mice with the minimal toxicity, we fed the mice with CBD at the dose of 3 and 10 mg/kg body weight per day by oral gavage for 7 days on chow diet (3 mice/group). CBD was first dissolved in DMSO and next mixed with corn oil (Fisher Science Education, S25271) to make the oral gavage mixture, 100 µL of which was used to treat each mouse per day. Sample size was determined based on the differences in means and standard deviation of intestinal *CYP3A11* gene expression levels between control and tributyl citrate treated WT mice (28) to estimate the minimum number of animals required to significantly differentiate two groups at a P value of 0.05 and a Power level of 90% (41, 42). The LaMorte’s Power Calculator Microsoft excel spreadsheet was downloaded from the web site (43). Blood and major tissues were collected. Our preliminary data showed that CBD at the dose of 10 mg/kg, but not 3 mg/kg, could significantly activate hepatic PXR, although neither dose affected the major organ weights (**Supplementary Figure 1**). Therefore, CBD at the dose of 10 mg/kg/day was used for our study.

To study if the potential CBD roles were PXR-dependent, we recruited an established specific PXR antagonist *trans*-resveratrol (RES) which is a natural stilbenoid found in fruits (44). Various doses of RES (from 22.5 to 2500 mg/kg BW daily) have been used in different studies in mice (45–47). RES at 45 mg/kg significantly improved osteoarthritis symptoms in mice (46), and the diet-induced obesity mice fed RES by gavage (75 mg/kg) did not show hepatic toxicity (47). To establish the optimal dose of PXR antagonist RES, we fed the mice (3 mice/group) by oral gavage with CBD at a dose of 10 mg/kg BW daily together with the two different doses of RES (45 and 75 mg/kg/day). The compounds were dissolved in DMSO and next mixed with corn oil to make the oral gavage mixture, 100 µL of which was used to treat each mouse per day. The preliminary data suggested that either dose could inhibit the PXR activation by CBD treatment in liver (**Supplementary Figure 2**). Thus, RES at the dose of 45 mg/kg/day was utilized in our study.

Groups of 8-week-old male C57BL/6 wildtype mice were orally gavaged with CBD at a dose of 10 mg/kg BW per day with or without PXR specific antagonist RES (45 mg/kg/day) for 7 days on a semisynthetic low-fat AIN76 diet containing 0.02% cholesterol (Research Diet, D00110804C). The compounds were dissolved in DMSO and mixed with corn oil to make the oral gavage mixture (100 µL of gavage mixture/mouse per day). The AIN76 diet has been previously used for dyslipidemia and atherosclerosis research in mice (36, 48). Sample size was determined based on the differences in means and standard deviation of plasma total cholesterol levels between control and dicyclohexyl phthalate treated WT mice on AIN76 diet to estimate the minimum number of animals required to significantly differentiate two groups at a P value of 0.05 and a Power level of 90% (41, 42). The LaMorte’s Power Calculator Microsoft excel spreadsheet was downloaded from the web site (43). On the day of euthanasia, mice (6 mice/group) were fasted for 6 hours following the feeding cycle. After the bodyweight was measured, mice were then anesthetized with ketamine/xylazine (100/10 mg/kg) by intraperitoneal injection and dissected to open the peritoneum and chest cavity. Blood was collected by left ventricle puncture, and the circulatory system was perfused by injecting saline into the left ventricle after nicking the right atrium. The major organs were collected and weighted. Mice were housed in the animal facilities with temperature-controlled rooms and 12-hour light/dark cycle. All animal studies were performed in compliance with approved protocols by Institutional Animal Care and Use Committees of University of Nebraska at Kearney.

### Plasma analysis

Plasma total cholesterol and triglyceride concentrations were determined enzymatically by colorimetric methods as per the manufacturer’s protocol (Fujifilm, Cholesterol 999–02601 and triglyceride 994–02891). The blood was collected in tubes with Heparin as an anticoagulant and centrifuged at 1500 x g at 4°C for 15 min. Plasma was collected and stored at -80°C. The HDL and LDL/VLDL lipoprotein fractions were quantified using HDL and LDL/VLDL Cholesterol Assay Kit as per the manufacturer’s protocol (Abcam, ab65390).

### RNA isolation and real-time quantitative PCR analysis

Total RNA was isolated from mouse tissues or cells using TRIzol Reagent following the manufacture’s protocol (Thermo Fisher Scientific, 15596026). RNA concentrations and quality were evaluated by NanoDrop Spectrophotometers (Thermo Fisher Scientific, ND-ONE-W). Two micrograms of total RNA were reverse-transcribed into cDNA using SuperScript III reverse transcriptase (Invitrogen, 18080093). Quantitative real-time PCR (QPCR) was performed using PowerUp SYBR Green Master Mix Green Supermix (Thermo Fisher Scientific, A25777) on a CFX Real-Time PCR Instrument (Bio-Rad, 184–5096) as per the manufacturer-supplied protocol. The gene-specific primers were purchased from Integrated DNA Technologies, and the sequences of primer sets were listed in **Supplementary Table 2.** For each biological sample, two technical replicate cycle threshold (Ct) values were collected and averaged. The mean Ct values were normalized to glyceraldehyde-3-phosphate dehydrogenase (*GAPDH*), and the relative mRNA expression levels were calculated using the comparative ΔΔCt method (49). The relative gene expression was presented as mean fold change over control samples.

### Cholesterol uptake assay

To investigate the roles of CBD-induced PXR activation in intestinal cholesterol uptake, human intestinal LS180 cells were seeded in a black flat bottom 96 well plate and propagated to 50% confluence. The cholesterol uptake was analyzed using Abcam Cholesterol Uptake Assay Kit following the manufacturer’s manual (Abcam, ab236212). Previous study suggested that 50 µM of RES treatment effectively reduced the rifampicin-induced PXR activity in hPXR-overexpressed human intestinal LS174T cells without affecting the cell survival (44). Thus, we hypothesized 50 µM of RES in our study could effectively inhibit the potential CBD-induced cholesterol uptake by prohibiting PXR signaling. Briefly, LS180 cells were treated with 10 µM of CBD or DMSO control in the presence/absence of 50 µM of PXR antagonism RES in serum-free culture medium containing 20 μg/mL of green fluorescent protein (GFP)-tagged Cholesterol for 24 hours. At the end of the experiment, the degree of cholesterol uptake was analyzed using Synergy H1 Hybrid Reader at Ex/Em = 485/535 nm. The result was displayed as the fold activation of relative fluorescence units compared to DMSO vehicle.

### Statistical analysis

All data are presented as the mean ± SEM and the individual data points are also shown in the figures. Individual pairwise comparisons were analyzed by two-sample, two-tailed Student’s t-test. One-way analysis of variance (ANOVA) was used when multiple comparisons were made, followed by Dunnett’s t test for multiple comparisons to a control. Two-way ANOVA was used when multiple comparisons were made followed by a Bonferroni multiple comparisons test. N numbers are listed in figure legends and P<0.05 was regarded as significant. Two-way ANOVA were done using SigmaPlot 13.0. The other statistics were analyzed using GraphPad Prism.

## Results

### Evaluate CBD as a potential PXR agonist

**Figure 1:**
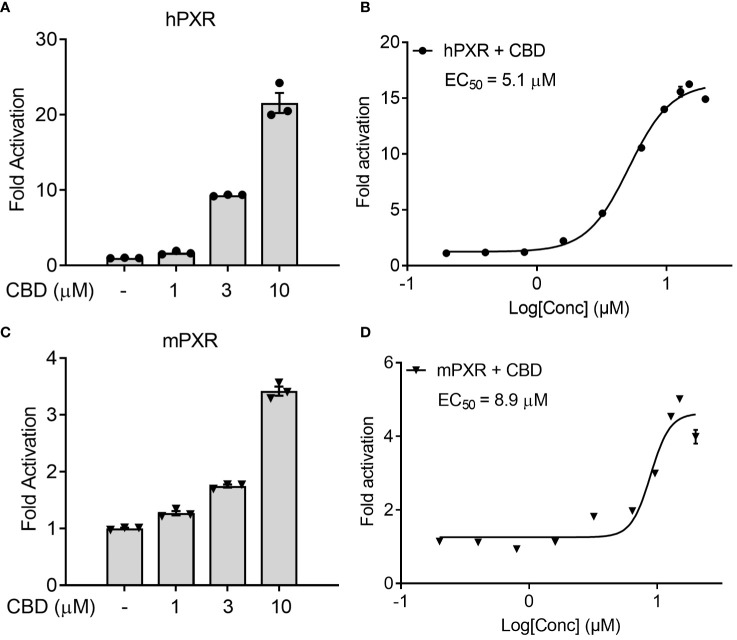
The effects of CBD on PXR activity in human hepatic HepG2 cells using transfection assay. **(A, B)** Human HepG2 hepatic cells were transfected with full-length hPXR plasmid together with hPXR reporter CYP3A4-luc and β-galactosidase (β-gal) control plasmids. Cells were then treated with CBD **(A)** at the indicated concentrations or **(B)** at the doses from 0.2 to 20 µM for 24 hours (n=3). **(C, D)** Human HepG2 hepatic cells were transfected with full-length mPXR plasmid and mPXR reporter (CYP3A2)_3_-luc together with β-gal control plasmids. Cells were then treated with CBD **(C)** at the indicated concentrations or **(D)** at the doses from 0.2 to 20 µM for 24 hours (n=3). Reporter gene activity was normalized to the β-gal transfection controls and the results were normalized to Relative Light Unit (RLU) per OD_420_ β-gal per minute to facilitate comparisons between plates. Fold activation was calculated relative to vehicle DMSO controls. Error bars represent ± SEM.

We first used the cell-based transfection assay in human HepG2 hepatic cells to test the ability of CBD to activate human PXR (hPXR) or mouse PXR (mPXR). CBD induced PXR reporter activities in a dose-dependent manner with higher activity in hPXR reporter than mPXR reporter (**Figures 1A, C**). Dose response curve analysis suggested that the EC_50_ for CBD activation of hPXR-mediated CYP3A4 promoter activity was 5.1 μM (**Figure 1B**), and that the EC_50_ for mPXR-mediated CYP3A2 promoter activity was 8.9 μM (**Figure 1D**).

**Figure 2:**
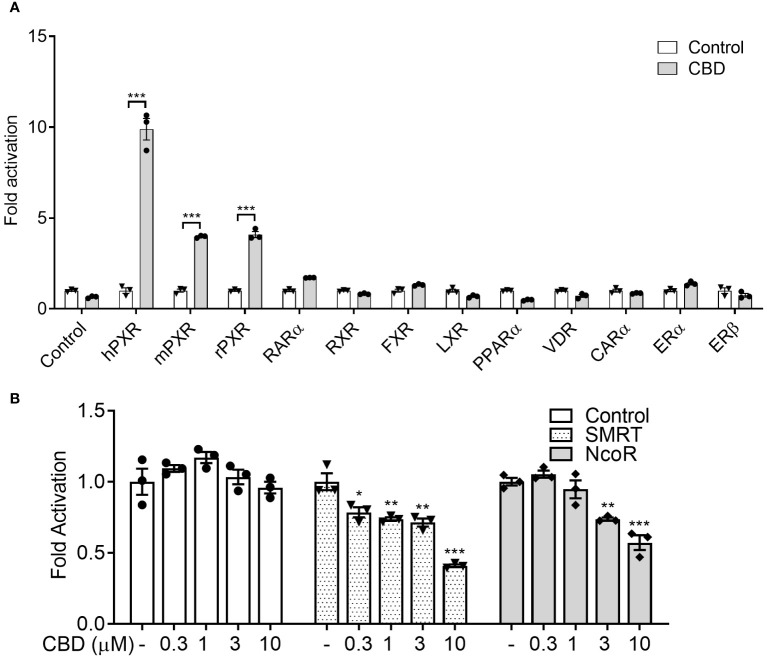
Evaluate if CBD is a selective PXR agonist. **(A)** HepG2 cells were transfected with a GAL4 reporter and a series of GAL4 plasmids in which the GAL4 DNA binding domain **(DBD)** is linked to the indicated nuclear receptor ligand binding domain (LBD). Cells were treated with DMSO control or 10 µM CBD for 24 hours (n=3, two-sample, two-tailed Student’s t-test, ***P<0.001 compared to control group). **(B)** HepG2 cells were transfected with a GAL4 reporter, VP16-hPXR vector, and expression vector for GAL4 DBD or GAL4 DBD linked to the receptor interaction domains of PXR co-repressors (GAL4-SMRT or GAL4-NCoR). Cells were treated with DMSO vehicle control or CBD at the indicated concentrations for 24 hours (n=3, one-way ANOVA, Dunnett’s t test for multiple comparisons to control, *P<0.05, **P<0.01, and ***P<0.001). Reporter gene activity was normalized to the β-gal transfection controls and the results were normalized to Relative Light Unit (RLU) per OD_420_ β-gal per minute to facilitate comparisons between plates. Fold activation was calculated relative to vehicle DMSO controls. Error bars represent ± SEM.

To determine whether CBD was a PXR-specific agonist, we examined the ability of CBD to activate a number of other nuclear receptors, including rat PXR (rPXR), retinoid X receptor (RXR), human retinoid acid receptor (RAR)α, constitutive androstane receptor (CAR)α, liver X receptor (LXR)α, farnesoid X receptor (FXR), peroxisome proliferator-activated receptor (PPAR)α, vitamin D receptor (VDR), Estrogen Receptor (ER)α, and ERβ. Except for PXRs, CBD had little effect on activation of the other tested nuclear receptors, suggesting that CBD was a selective agonist of PXR with higher activities on human PXR than rodents PXRs (**Figure 2A**). Thus, we next sought to explore CBD/PXR agonism. Since nuclear co-regulators play important roles in nuclear receptor activation, we evaluated if CBD altered the interaction between PXR and nuclear co-repressors using mammalian two-hybrid assay. Unliganded hPXR interacted with the co-repressors silencing mediator of retinoid and thyroid hormone (SMRT) and nuclear receptor co-repressor (NCoR) in the absence of CBD (**Figure 2B**). CBD, in a dose-dependent way, promoted the dissociation of hPXR from SMRT or NCoR (**Figure 2B**), resulting in the induced hPXR transcriptional activation.

### Key amino acid residues of hPXR LBD required for CBD’s agonistic activity

**Figure 3:**
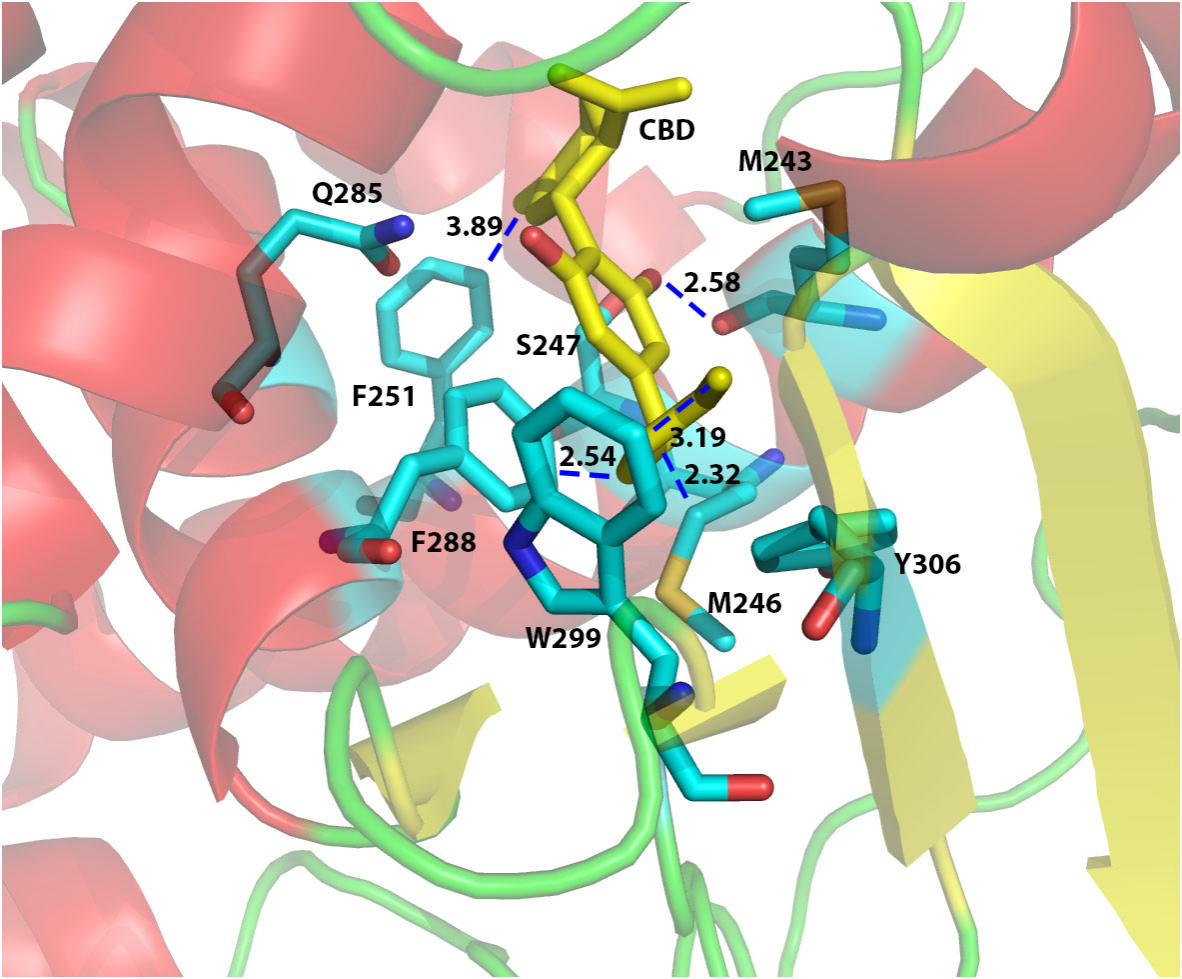
Computational docking of CBD to the ligand binding pocket of human PXR. The predicted residues required for CBD (yellow) binding were highlighted in cyan color. The distances (in Å) between the CBD and the residues in hPXR (4X1F wt) ligand binding pocket were indicated to understand the hydrogen bond interaction, aromatic-aromatic interaction, or hydrophobic interaction.

In order to investigate the binding interactions between CBD and hPXR, we docked CBD to the hPXR (4X1F wt model) using the Glide Dock program in the Schrödinger software suite. The docking results suggested that eight residues within hPXR LBD pocket could possibly be responsible for the agonistic binding of the CBD (**Figure 3**). For example, the residue Phe251 formed Van der Waals interaction (favorable hydrophobic interaction) between the aromatic ring on the Phe251 and the cyclohexene on the CBD with the distance of 3.89 Å (**Figure 3**). In addition, the residue Phe288 formed a π-π stacking interaction with the aromatic ring of the CBD. Both Phe288 and Trp299 provided Van der Waals interactions with the hydrophobic pentyl group of the CBD at the distances of 2.54 Å and 3.19 Å, respectively (**Figure 3**). To further evaluate the roles of these residues in CBD binding, we computationally mutated these residues and docked CBD to these mutant proteins. The relative Glide Scores ΔΔG of the mutated residues Met246Ala, Ser247Leu, Phe251Leu, Phe288Ala, Trp299Leu, and Tyr306Phe were above zero suggesting that they were possibly required for CBD binding (**Supplementary Table 3**). On the contrary, the relative Glide Score of Gln285Ala was below zero which suggested that Gln285 was independent of hPXR/CBD binding. Although the residue Met243 formed a hydrogen bond with the phenol group on the CBD (the distance between two oxygen atoms that formed H-bond was 2.58 Å) (**Figure 3**), the relative Glide Score ΔΔG of Met243Ala was zero (**Supplementary Table 3**), implying that Met243 was not required in the interaction of hPXR and CBD. The hydrogen bond of Met243 with CBD was mediated via the main chain carbonyl group, which was maintained when Met243 was mutated to Alanine, thus contributing to the ΔΔG of Met243Ala being zero.

**Figure 4:**
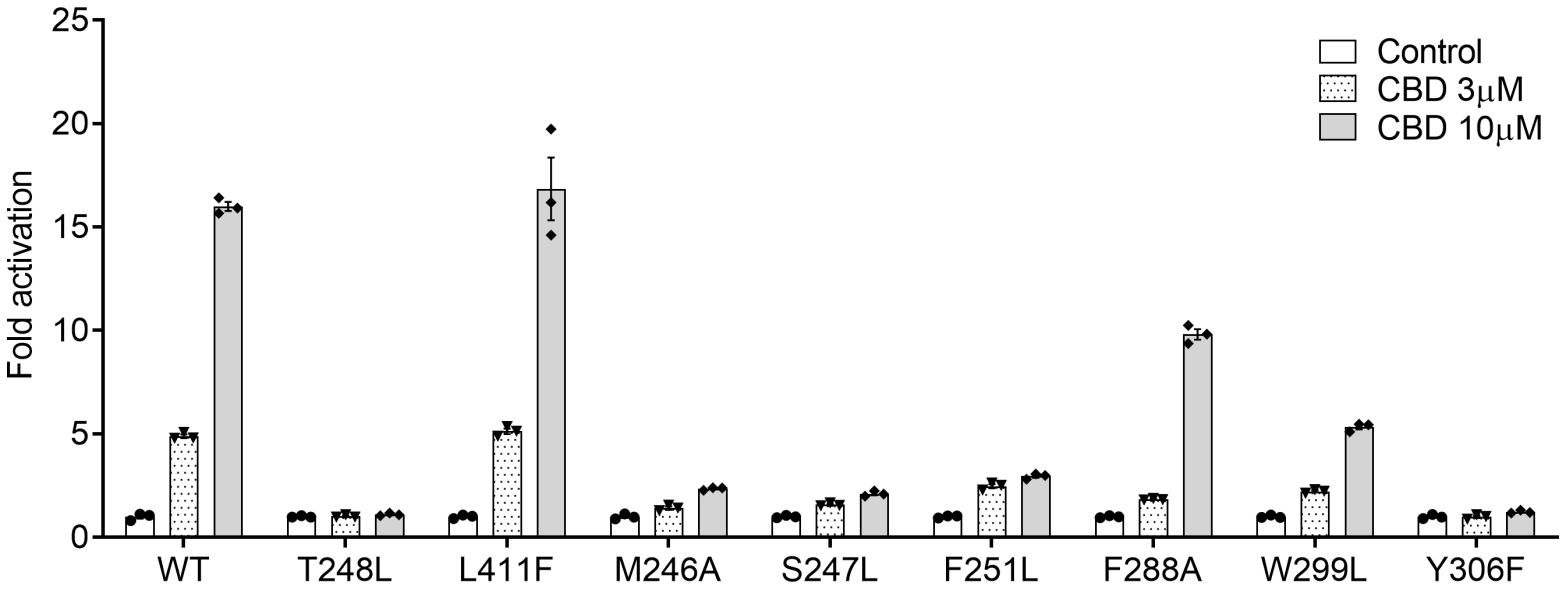
Key residues of PXR LBD required for CBD’s agonistic activity. HepG2 cells were co-transfected with a full-length hPXR WT plasmid or mutant plasmids as indicated, together with CYP3A4-luc reporter and β-gal plasmid. After transfection, cells were treated with control medium or medium containing 3 or 10 μM of CBD for 24 hours. Reporter gene activity was normalized to the β-gal transfection controls and the results were normalized to Relative Light Unit (RLU) per OD_420_ β-gal per minute to facilitate comparisons between plates. Fold activation was calculated relative to vehicle DMSO controls. Error bars represent ± SEM.

Based on the results from the docking study, we mutated the key amino acids within hPXR LBD required for CBD’s agonistic activity using the site-directed mutagenesis and cell-based transfection assays. Leu411, a predicted residue within PXR’s ligand binding pocket which didn’t interact with CBD, was used as a negative control in this study. Thr248, a known key amino acid important for PXR/co-activator interaction, was mutated as the positive control (50). As expected, Leu411Phe mutation had no impacts on CBD activity, whereas Thr248Leu mutation completely blocked the activity of CBD (**Figure 4**). Our results suggested that CBD’s agonistic activity was abolished by the mutations of Met246Ala, Ser247Leu, Phe251Leu, and Tyr306Phe, and was weakened by Phe288Ala and Trp299Leu mutations (**Figure 4**). Taken together, our data identified the key amino acid residues within PXR’s binding pocket that are necessary for the agonistic effects of CBD utilizing the docking study together with site-directed mutagenesis analysis.

### Effects of CBD on plasma lipid levels in wildtype mice

In a recent study CBD was identified to increase lipid peroxidation and free fatty acid levels in rats at a dose of 10 mg/kg daily (14). To determine the optimal CBD dose that could induce the PXR in mice with the minimal toxicity, we first fed the C57BL/6 wildtype (WT) mice with CBD at the dose of 3 or 10 mg/kg body weight (BW) per day by oral gavage for seven days. QPCR analysis in liver showed that the expression level of known mouse PXR target gene *CYP3A11* was induced at the dose of 10 mg/kg BW but not at 3 mg/kg BW (**Supplementary Figure 1A**). The weights of major organs, including liver, spleen, and kidney, were not affected by CBD exposure at either dose (**Supplementary Figures 1B-D**). These data suggested that the dose of 10 mg/kg BW was optimal to activate PXR signaling without causing toxicity to the major organs. To study if the potential CBD roles were PXR-dependent, we recruited an established specific PXR antagonist RES (44). To establish the optimal dose of PXR antagonist RES, we fed the mice by oral gavage with CBD at a dose of 10 mg/kg BW daily together with the two different doses of RES (45 and 75 mg/kg/day). QPCR results suggested that the expression level of hepatic PXR target gene *CYP3A11* was decreased by RES at the dose of either 45 or 75 mg/kg BW per day (**Supplementary Figure 2**).

**Figure 5:**
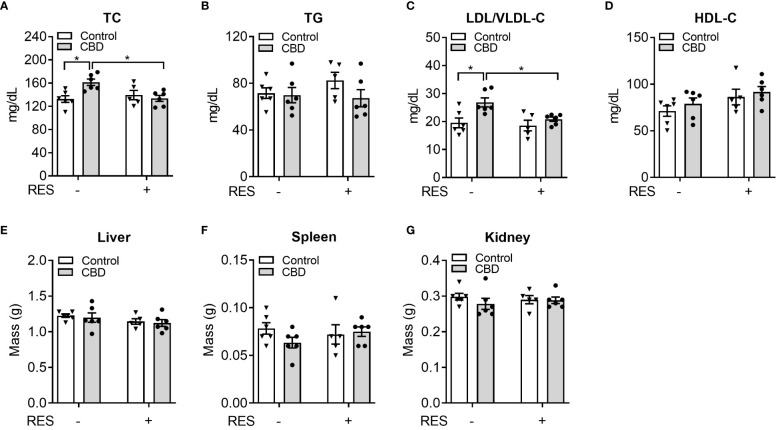
The impacts of CBD exposure on plasma lipid levels in wildtype mice. Eight-week-old male WT mice were treated with vehicle control or CBD (10 mg/kg/day) and/or PXR specific antagonist RES (45 mg/kg/day) by oral gavage for 1 week on AIN76 diet. Fasting plasma total cholesterol **(A)** and triglyceride **(B)** levels were measured by enzymatically colorimetric methods (n=5–6, two-way ANOVA, Bonferroni multiple comparisons test for multiple comparisons, *P<0.05). **(C, D)** Lipoprotein fractions (LDL/VLDL and HDL) were isolated, and the cholesterol levels of each fraction were measured (n=5–6, two-way ANOVA, Bonferroni multiple comparisons test for multiple comparisons, *P<0.05). The major organs were weighed at anesthesia, including liver **(E)**, spleen **(F)**, and kidney **(G)**. Error bars represent ± SEM.

To investigate whether CBD exposure could change plasma lipid levels *in vivo*, we next used CBD (10 mg/kg/day) together with RES (45 mg/kg/day) to treat C57BL/6 WT male mice for a week on AIN76 diet which was previously used for dyslipidemia and atherosclerosis research in mice (36, 48). Short-term exposure to CBD led to significantly higher plasma total cholesterol levels (**Figure 5A**) but did not affect plasma triglyceride levels (**Figure 5B**) or major organ weight (**Figures 5E**-G). Interestingly, this increased total cholesterol level was abolished in the presence of PXR antagonist RES, suggesting a PXR-dependent way (**Figure 5A**). Moreover, WT mice treated with CBD had significantly higher atherogenic VLDL and LDL cholesterol levels than control mice, but displayed unchanged lipoprotein cholesterol levels in RES treated mice, suggesting that CBD could affect cholesterol levels through PXR signaling pathway (**Figure 5C**). However, the HDL cholesterol levels in the plasma were not altered by CBD treatment (**Figure 5D**). These data suggested that short-term exposure to CBD could increase the atherogenic cholesterol levels in plasma through PXR signaling.

### Impacts of CBD exposure on intestinal lipogenic gene expression in WT mice

**Figure 6:**
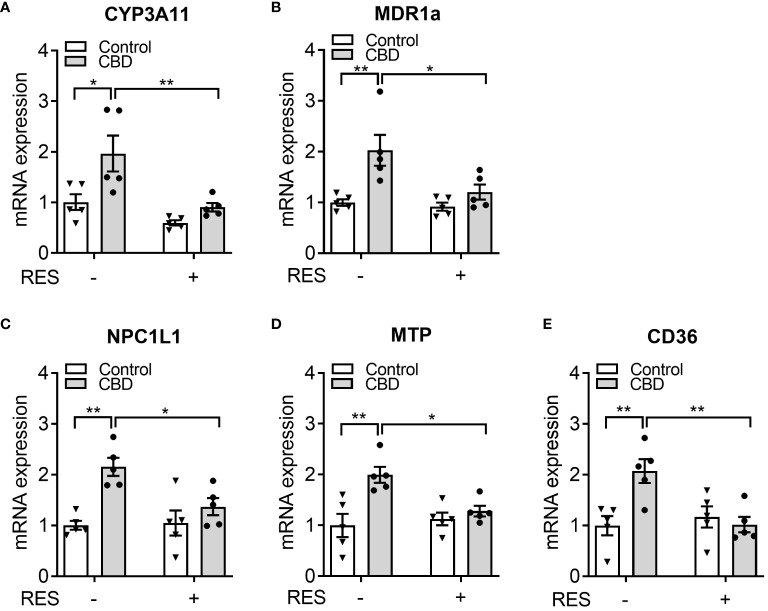
The effects of CBD exposure on intestinal lipogenic gene expression in WT mice. Male WT mice at the age of 8 weeks old were administrated with CBD (10 mg/kg/day) and/or RES (45 mg/kg/day) or vehicle control by oral gavage for 7 days on AIN76 diet. **(A, B)** Intestinal expression of PXR target genes was measured by QPCR (n=5, two-way ANOVA, Bonferroni multiple comparisons test for multiple comparisons, *P<0.05 and **P<0.01). **(C-E)** The gene expression of key intestinal cholesterol transporters was analyzed by QPCR (n=5, two-way ANOVA, Bonferroni multiple comparisons test for multiple comparisons, *P<0.05 and **P<0.01). Error bars represent ± SEM.

To confirm if CBD increased circulating cholesterol levels via PXR signaling, the known PXR target genes were analyzed by QPCR assay in the small intestines from C57BL/6 WT mice orally fed CBD (10 mg/kg/day) with/without RES (45 mg/kg/day) for one week. QPCR results showed that the mRNA expression levels of two PXR target genes, *CYP3A11* and *MDR1a*, were induced by CBD exposure in the absence of RES but not in RES fed mice, which suggested the activation of intestinal PXR pathway by CBD treatment *in vivo* (**Figures 6A, B**). We next examined the influence of CBD on the intestinal genes, which regulate lipid homeostasis and are also the direct transcriptional targets of PXR. The key intestinal cholesterol transporters Niemann-Pick C1-Like 1 (*NPC1L1*) (28), microsomal triglyceride transfer protein (*MTP*) (23), and cluster of differentiation 36 (*CD36*) (26, 51) had higher mRNA expression levels with CBD treatment in WT mice but not in the PXR-inhibited mice (**Figures 6C–E**). Taken together, exposure to CBD increased the atherogenic cholesterol levels which was partially caused by the induced expression levels of the key intestinal PXR-regulated lipogenic genes (**Supplementary Figure 3**).

### Assess the potential roles of CBD in cholesterol uptake by human intestinal cells

**Figure 7:**
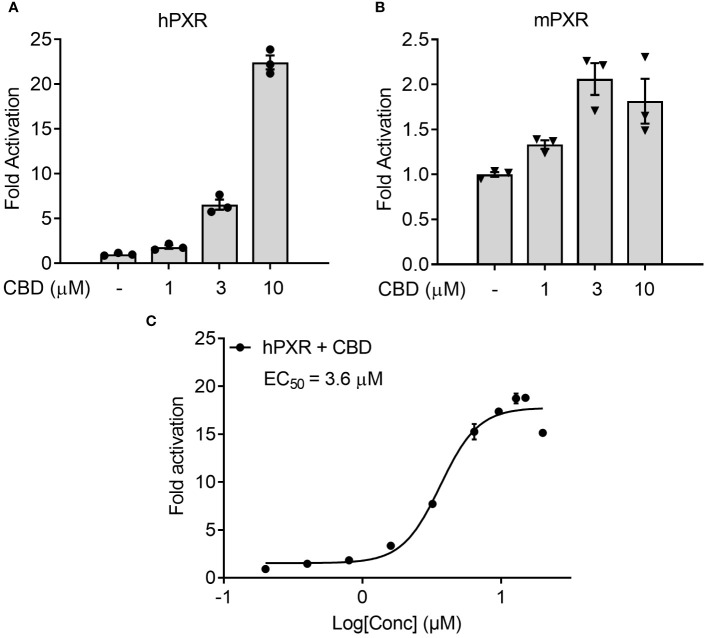
The effects of CBD on PXR activity in human intestinal LS180 cells using transfection assay. **(A, C)** Human LS180 intestinal cells were transfected with full-length hPXR plasmid together with hPXR reporter CYP3A4-luc and β-galactosidase (β-gal) control plasmids. Cells were then treated with CBD **(A)** at the indicated concentrations or **(C)** at the doses from 0.2 to 20 µM for 24 hours (n=3). **(B)** Human LS180 cells were transfected with full-length mPXR plasmid and mPXR reporter (CYP3A2)_3_-luc together with β-gal control plasmids. Cells were then treated with CBD at the indicated concentrations for 24 hours (n=3). Reporter gene activity was normalized to the β-gal transfection controls and the results were normalized to Relative Light Unit (RLU) per OD_420_ β-gal per minute to facilitate comparisons between plates. Fold activation was calculated relative to vehicle DMSO controls. Error bars represent ± SEM.

To examine the possible mechanisms by which CBD increased the plasma cholesterol levels, human intestinal LS180 cells were used to study whether CBD treatment could alter the cholesterol uptake through intestinal PXR signaling. We first performed the transfection assay to evaluate the capability of CBD to activate hPXR or mPXR in mammalian LS180 cells. CBD induced hPXR reporter activities in a concentration-dependent manner (**Figure 7A**). Dose response curve analysis suggested that the EC_50_ for CBD activation of hPXR-mediated CYP3A4 promoter activity was 3.6 μM (**Figure 7C**). Compared with hPXR reporter activity (6-fold activation at 3 µM of CBD treatment), mPXR-mediated CYP3A2 promoter activity was weaker (2-fold activation at 3 µM of CBD treatment) and trended down when the CBD dose was increased to 10 µM (**Figures 7A, B**). This was consistent with what we found in Human hepatic HepG2 cells (**Figures 1A, C**), suggesting that CBD is a PXR agonist with higher activities on human PXR than mouse PXR.

**Figure 8:**
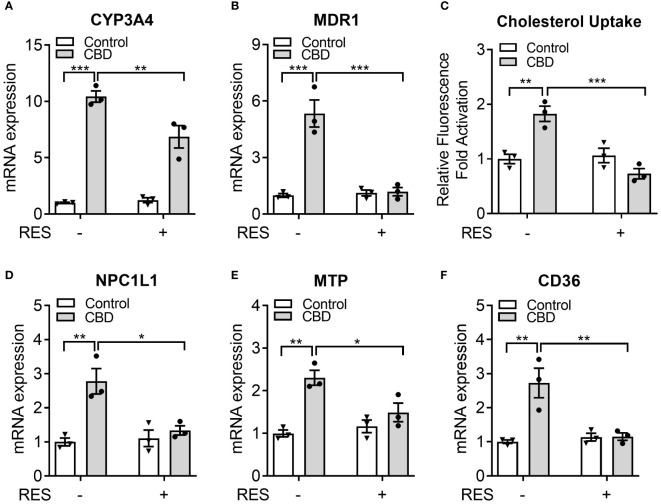
The impacts of CBD exposure on cholesterol uptake by human intestinal LS180 cells. Human LS180 cells were treated with vehicle control or 10 µM of CBD in the presence/absence of PXR antagonist RES (50 µM) in serum-free culture media for 24 hours. **(A, B)** Intestinal expression of PXR target genes was measured by QPCR (n=3, two-way ANOVA, Bonferroni multiple comparisons test for multiple comparisons, *P<0.05, **P<0.01 and ***P<0.001). **(C)** GFP-tagged cholesterol was used to cotreat the cells for 24 hours. The degree of cholesterol uptake was quantified at Ex/Em = 485/535 nm. The result was displayed as the fold activation of relative fluorescence units compared to DMSO vehicle (n=3, two-way ANOVA, Bonferroni multiple comparisons test for multiple comparisons, **P<0.01 and ***P<0.001). **(D-F)** The gene expression of key intestinal cholesterol transporters was analyzed by QPCR (n=3, two-way ANOVA, Bonferroni multiple comparisons test for multiple comparisons, *P<0.05 and **P<0.01). Error bars represent ± SEM.

Previous study suggested that 50 µM of RES treatment effectively reduced the rifampicin-induced PXR activity in hPXR-overexpressed human intestinal LS174T cells without affecting the cell survival (44). To determine if RES could inhibit the potential PXR activation by CBD in human intestinal cells, LS180 cells were treated with 10 µM of CBD and/or 50 µM of RES for 24 hours. QPCR analysis showed that the expression levels of PXR target genes *CYP3A4* and *MDR1* were increased by CBD treatment and inhibited by RES cotreatment (**Figures 8A, B**), which suggested that PXR activation by CBD in human intestinal LS180 cells could be inhibited by RES. To evaluate whether CBD affected cholesterol uptake by human intestinal cells, the GFP-labeled cholesterol was used to incubate LS180 cells for 24 hours. Interestingly, the cholesterol uptake was ascended in the CBD treated cells but not in the RES/CBD cotreated cells, suggesting that CBD could increase cholesterol uptake by human intestinal cells through PXR signaling (**Figure 8C**). Consistently, QPCR analysis showed that the gene expression levels of cholesterol transporters, *NPC1L1*, *MTP*, and *CD36*, were induced by CBD treatment in LS180 cells but not in RES-incubated cells (**Figures 8D–F**). In sum, our data suggested that CBD exposure upregulated the cholesterol uptake by human intestinal cells, which could partially result from the increased expression levels of PXR-mediated intestinal cholesterol transporters (**Supplementary Figure 3**).

## Discussion

CBD, as a non-intoxicating substance in cannabis, has the broad potential to treat epilepsy, neurodegenerative diseases, neuropsychiatric disorders, gastrointestinal disorders, rheumatic diseases, and graft versus host disease (52–55). However, it is still unclear whether CBD has adverse cardiovascular effects and which nuclear receptor(s) CBD can activate for the cellular signal transduction, since the affinity of CBD to cannabinoid receptors is weak (15). In the current study, to the best of our knowledge, our study is the first to identify CBD as a PXR agonist and to explore the key amino acid residues within human PXR LBD which interact with CBD. We also found that short term CBD exposure could increase the atherogenic cholesterol levels in plasma, which could be partially caused by the ascended intestinal cholesterol uptake through PXR signaling.

The effects of CBD on cardiovascular parameters are controversial. CBD not only decreased the blood pressure in anesthetized mice and rats (56, 57) but also increased blood pressure and heart rate in both anaesthetized dogs (58) and in conscious rats (59), suggesting that CBD has minimal influences on the arterial blood pressure and heart rate in the animals under physiological condition (60). The activation of central cannabinoid receptors increases blood pressure, whereas the peripheral cannabinoid receptors innervate the vascular resistance responsible for hypotensive effects of cannabinoids. Cannabinoids, intriguingly, cause not only vasodilation but also vasoconstriction in isolated blood vessels or perfused vascular beds (61, 62). Except for cannabinoid receptors, cannabinoids can activate other receptors in both cardiovascular system and the nervous system to affect cardiovascular function. For instance, cannabinoids were found to activate an endothelial cannabinoid receptor G protein-coupled receptor-18 in both peripheral blood vessels and central rostral ventrolateral medulla, leading to vasorelaxation and hypotension (63, 64). Therefore, CBD was suggested to have therapeutic potential to treat stroke and myocardial infarction because of the vasodilatory and neuroprotective properties (65, 66), however, the lifetime myocardial infarction odds were increased by up to 8% in cannabis users (67). It is possible that CBD has multidirectional impacts on the cardiovascular system considering its complex mechanism of action. Although associations between CBD and cardiovascular diseases have begun to emerge, the underlying mechanisms remain elusive.

The nuclear receptor (NR) superfamily consists of transcriptional regulators that control the assembly of the basal machinery and affect target gene expression levels. The large ligand binding domain of PXR binds to a variety of structurally distinct ligands to regulate PXR’s transcriptional activity (68). To date, no report has suggested whether CBD could activate PXR in humans or animals. By use of transfection assays in both human hepatic and intestinal cells, CBD was found to induce PXR activities of human PXR reporter to a greater degree than that of mouse PXR reporter (**Figures 1**, **7**). Upon ligand binding, the AF-2 region of the LBD adopts a conformation that dissociates corepressor proteins to upregulate target gene expression (69). We also used the mammalian two-hybrid assay to evaluate the effects of CBD on hPXR and coregulator interaction, which is a critical part of nuclear receptor signaling pathways. CBD promoted the specific dissociation of PXR from its co-repressors NCoR and SMRT (**Figure 2B**). Moreover, CBD was suggested as a PXR selective agonist since it activated only PXRs rather than the other NRs we tested (**Figure 2A**). Further, based on our data from computational docking studies and site-directed mutagenesis assay, we deduced the required structure below for CBD/PXR interaction. Three highly conserved residues Phe288, Trp299, and Tyr306 lined a hydrophobic region within the PXR ligand binding pocket where the residues Met246 and Ser247 interacted with CBD through essential hydrogen bonds (50). Specifically, the aromatic ring on the residue Phe251 formed the hydrophobic interaction with the cyclohexene on the CBD to stabilize the binding between PXR and CBD. Our data is the first to link CBD to pro-atherosclerotic PXR signaling pathway and to identify which amino acid residues are critical for CBD interaction with PXR ligand binding pocket.

PXR functions as a xenobiotic sensor that activates the expression of genes required for xenobiotic metabolism in the liver and intestine, including cytochrome P450s (*CYP*), conjugating enzymes (e.g., glutathione transferase (*GST*)), and ABC family transporters (e.g., multidrug resistance 1 (*MDR1*)) (19, 21). In addition to the important roles in xenobiotic metabolism, PXR signaling has been associated with lipid homeostasis (20). For example, the chronic activation of PXR by feeding mice the potent PXR ligand pregnenolone 16α-carbonitrile (PCN) led to increased plasma total and LDL cholesterol levels in WT mice, but not in PXR deficient mice (24). Activation of PXR can also increase plasma total cholesterol and VLDL levels in apolipoprotein E (ApoE)-Leiden mice, which have a human-like lipoprotein distribution pattern (25). The impacts of CBD on human cardiovascular system depend on the delivery method, the dose (70), and the duration of administration (71). In a recent study after CBD was injected at a dose of 10 mg/kg daily for 14 days, the rats showed increased lipid peroxidation and free fatty acid levels (14). In the present study, when CBD at the dose of 10 mg/kg was used to feed mice, the expression level of PXR target gene *CYP3A11* was increased in both liver and intestine, suggesting the effective activation of PXR signaling. Indeed, the plasma total cholesterol level and atherogenic LDL/VLDL cholesterol level were upregulated by CBD treatment, which was abolished by PXR antagonist RES treatment (**Figure 5**). These data suggested that CBD potentially induced hypercholesterolemia in mice by activating PXR.

To maintain the lipid homeostasis the intestinal lipid transportation plays an important role. In human intestinal LS180 cells, CBD incubation upregulated the GFP-tagged cholesterol uptake, which was blocked by the coincubation of PXR inhibitor RES (**Figure 8C**). PXR has been shown to regulate multiple intestinal and hepatic genes involved in lipid homeostasis in different animal models (20, 21, 25, 26, 28). Although the LBD of PXR displays species-specific properties, the DNA-binding domain (DBD) of PXR is conserved between humans and rodents (20). Our data showed that CBD mediated PXR activation significantly increased the expression of three key lipogenic genes in both human LS180 cells and WT mice, including *NPC1L1*, *MTP*, and *CD36*, which are direct transcriptional targets of PXR. The core intestinal cholesterol transporter NPC1L1 has been used as a clinical target of cholesterol-lowering drug Ezetimibe that inhibits cholesterol absorption (72). The overexpression of intestinal MTP, which was important for lipid absorption and lipoprotein assembly (73), induced hyperlipidemia and atherosclerosis in mice (74, 75). Besides, the membrane protein CD36 played a key role in cholesterol uptake by the proximal but not distal intestine (51).

In this study we investigated the effects of short-term exposure to CBD on plasma lipid levels in male mice, however further studies are needed to reveal whether CBD alters lipid profiles in female mice and how long-term CBD exposure could affect atherosclerotic development. The chronic activation of PXR by ligands could increase the lipid uptake by macrophage to promote the foam cell formation leading to atherogenesis in hyperlipidemic ApoE^-/-^ mice (24, 48). CBD treated microglial cells displayed the upregulated levels of the cholesteryl esters synthesis enzyme sterol-O-acyl transferase (Soat2), sterol 27-hydroxylase (Cyp27a1), and lipid droplet-associated protein perilipin2 (Plin2), suggesting the possible regulation of CBD on cholesterol homeostasis (13). Hypercholesterolemia is a major risk factor for atherosclerosis (76), therefore, future studies are needed to understand how chronic exposure to CBD could change the functions of macrophage or other cell types to promote atherosclerosis. Furthermore, the activation of PXR by certain ligands also demonstrated the tissue-specific pattern. For example, an FDA-approved plasticizer Tributyl citrate can only activate intestinal PXR but not hepatic PXR (28), whereas a vitamin E family member Tocotrienols can upregulate PXR target gene *CYP3A4* in hepatocytes but not in intestinal cells (31). Thus, the tissue-specific PXR knockout mice models are necessary to evaluate the potential functions of CBD in different tissues, such as liver, intestine, and macrophage. It is possible that CBD may increase cardiovascular risk in humans via both dyslipidemia-dependent and -independent mechanisms. In summary, here we explore the potential molecular mechanisms by which exposure to CBD activates human PXR and increases the risk of dyslipidemia. Our data provide evidence to inform future risk assessment for CBD and reveal the novel mechanistic links between pharmaceutical chemicals and cardiovascular disease risk.

## Data availability statement

The original contributions presented in the study are included in the article/**Supplementary Material**. Further inquiries can be directed to the corresponding author.

## Ethics statement

The animal study was approved by Institutional Animal Care and Use Committees of University of Nebraska at Kearney. The study was conducted in accordance with the local legislation and institutional requirements.

## Author contributions

CB: Formal analysis, Investigation, Methodology, Visualization, Writing – original draft, Writing – review & editing. WK: Formal analysis, Investigation, Methodology, Writing – review & editing. HZ: Investigation, Methodology, Writing – original draft, Writing – review & editing. AK: Conceptualization, Investigation, Writing – review & editing. YS: Conceptualization, Data curation, Formal analysis, Funding acquisition, Methodology, Supervision, Writing – original draft, Writing – review & editing.
