# Supplementary material for: Cannabidiol promotes intestinal cholesterol uptake mediated by Pregnane X receptor
Source: Front Endocrinol (Lausanne). 2024 Jun 18;15:1398462. doi: 10.3389/fendo.2024.1398462 (PMC11217338; doi:10.3389/fendo.2024.1398462)
Supplement: Supplementary file 1 [file DataSheet_1.docx]

***Supplementary Material***

**Supplementary Figures and Tables**

**Supplementary Figure 1. The effects of CBD exposure on hepatic PXR target gene expression and organ weight in WT mice.** Male WT mice at the age of 8 weeks old were treated with vehicle control or CBD (3 or 10 mg/kg/day) by oral gavage for 7 days on chow diet (3 mice/group). **(A)** Hepatic expression of PXR target gene CYP3A11 was measured by QPCR (n=3, one-way ANOVA, Dunnett’s t test for multiple comparisons to control, *P<0.05). **(B-D)** The major organs were weighed at anesthesia, including liver, spleen, and kidney. Error bars represent ± SEM.

**Supplementary Figure 2. The impacts of RES on CBD-induced mRNA expression of hepatic PXR target gene CYP3A11 in WT mice.** Eight-week-old male WT mice were treated with vehicle control or CBD (10 mg/kg/day) with/without PXR specific antagonist RES (45 or 75mg/kg/day) by oral gavage for 1 week on chow diet (3 mice/group). Hepatic expression of PXR target gene CYP3A11 was measured by QPCR (n=3, one-way ANOVA, Dunnett’s t test for multiple comparisons to CBD (10 mg/kg/day) group, *P<0.05). Error bars represent ± SEM.

**Supplementary Figure 3. Schematic representation of the potential role of PXR in mediating the impact of CBD exposure on intestinal cholesterol uptake.** CBD activates human PXR by interacting with the key amino acid residues Met246, Ser247, Phe251, Phe288, Trp299, and Tyr306 within PXR’s ligand binding pocket. The activated PXR signaling by CBD induces the gene expression of key intestinal cholesterol transporters NPC1L1, MTP, and CD36, leading to the increased cholesterol uptake by enterocytes, which could result in the ascended plasma LDL/VLDL cholesterol levels in mice exposed to CBD. LBD, ligand binding domain; DBD, DNA binding domain; NPC1L1, Niemann-Pick C1-Like 1; MTP, microsomal triglyceride transfer protein; CD36, cluster of differentiation; C, cholesterol; LDL, low-density lipoprotein; VLDL, very-low-density lipoprotein.

**Supplementary Table 1. Primer sequences for site-directed mutagenesis**

| **Mutant** | **Primer sequences** |
| --- | --- |
| PXR-M246A | 5’-CTGCTGCCCCACATGGCTGACGCGTCAACCTACATGTTCAAAGGC-3’ |
|  | 5’-GCCTTTGAACATGTAGGTTGACGCGTCAGCCATGTGGGGCAGCAG-3’ |
| PXR-S247L | 5’-CTGCTGCCCCACATGGCTGACATGTTAACCTACATGTTCAAAGGC-3’ |
|  | 5’-GCCTTTGAACATGTAGGTTAACATGTCAGCCATGTGGGGCAGCAG-3’ |
| PXR-T248L | 5’-CACATGGCTGACATGTCACTCTACATGTTCAAAGGCATCATC-3’ |
|  | 5’-GATGATGCCTTTGAACATGTAGAGTGACATGTCAGCCATGTG-3’ |
| PXR-F251L | 5’-GACATGTCAACCTACATGTTAAAAGGCATCATCAGCTTTGC -3’ |
|  | 5’-GCAAAGCTGATGATGCCTTTTAACATGTAGGTTGACATGTC -3’ |
| PXR-F288A | 5’-CGAGCTGTGTCAACTGAGAGCCAACACAGTGTTCAACGCGG-3’ |
|  | 5’-CCGCGTTGAACACTGTGTTGGCTCTCAGTTGACACAGCTCG-3’ |
| PXR-W299L | 5’-CAACGCGGAGACTGGAACCTTGGAGTGTGGCCGGCTGTCC-3’ |
|  | 5’-GGACAGCCGGCCACACTCCAAGGTTCCAGTCTCCGCGTTG-3’ |
| PXR-Y306F | 5’-GGGAGTGTGGCCGGCTGTCCTTCTGCTTGGAAGACACTGCAGG-3’ |
|  | 5’-CCTGCAGTGTCTTCCAAGCAGAAGGACAGCCGGCCACACTCCC-3’ |
| PXR-L411F | 5’-GCTCAGCACACCCAGCGGTTCCTGCGCATCCAGGACATAC-3’ |
|  | 5’-GTATGTCCTGGATGCGCAGGAACCGCTGGGTGTGCTGAGC-3’ |
|  |  |
|  |  |
|  |  |
|  |  |

**Supplementary Table 2. Primer sequences for QPCR**

| **Genes** | **Primer sequences** |
| --- | --- |
| Mouse CYP3A11 | 5’-CAGCTTGGTGCTCCTCTACC-3’ |
|  | 5’-TCAAACAACCCCCATGTTTT-3’ |
| Mouse MDR1a | 5’-CCCCCGAGATTGACAGCTAC-3’ |
|  | 5’- ACTCCACTAAATTGCACATTTCCTTC-3’ |
| Mouse NPC1L1 | 5’-TTGCCTTGACCTCTGGCTTAG-3’ |
|  | 5’-AGGGCGGATGAATCTGTGC-3’ |
| Mouse MTP | 5’-TGAGCGGCTATACAAGCTCAC-3’ |
|  | 5’-CTGGAAGATGCTCTTCTCGC-3’ |
| Mouse CD36 | 5’-CAGTCGGAGACATGCT-3’ |
|  | 5’-CTCGGGGTCCTGAGTT-3’ |
| Mouse GAPDH | 5’-AACTTTGGCATTGTGGAAGG-3’ |
|  | 5’-GGATGCAGGGATGATGTTCT-3’ |
| Human CYP3A4 | 5’-GGCTTCATCCAATGGACTGCATAAAT-3’ |
|  | 5’-TCCCAAGTATAACACTCTACACAGACAA-3’ |
| Human MDR1 | 5’-CCCATCATTGCAATAGCAGG-3’ |
|  | 5’-GAGCATACATATGTTCAAACTTC-3’ |
| Human NPC1L1 | 5’-CTTCTACCAGCATAGCTTTGCC-3’ |
|  | 5’-AGAGCCATACACGCCACAC-3’ |
| Human MTP | 5’-ACAAGCTCACGTACTCCACTG-3’ |
|  | 5’-TCCTCCATAGTAAGGCCACATC-3’ |
| Human CD36 | 5’-GCAAAATCCACAGGAAGTGATG-3’ |
|  | 5’-GCTAGAAAACGAACTCTGTACGTATAAGG-3’ |
| Human GAPDH | 5’-GGCCTCCAAGGAGTAAGACC-3’ |
|  | 5’-AGGGGAGATTCAGTGTGGTG-3’ |

**Supplementary Table 3.** **The binding affinity scores, Glide Scores (ΔG) and relative Glide Scores (ΔΔG, kcal/mol, ΔΔG = ΔG_mut – ΔG_wt) for the 4X1F wt and mutant models of hPXR**

| Docking | WT | M243A | M246A | S247L | F251A | Q285A | F288A | W299L | Y306F |
| --- | --- | --- | --- | --- | --- | --- | --- | --- | --- |
| Docking Glide Score (ΔG) | -10.35 | -10.35 | -9.32 | -10.04 | -9.74 | -10.60 | -9.61 | -9.82 | -9.87 |
| Relative Glide Score (ΔΔG) |  | 0.00 | 1.03 | 0.30 | 0.61 | -0.25 | 0.73 | 0.52 | 0.47 |
